# The Duration of Breastfeeding and Its Association with Metabolic Syndrome among Obese Children

**DOI:** 10.1155/2015/731319

**Published:** 2015-07-30

**Authors:** Renata Yakubov, Erez Nadir, Roni Stein, Adi Klein-Kremer

**Affiliations:** ^1^Department of Pediatrics, Hillel Yaffe Medical Center, 38100 Hadera, Israel; ^2^Department of Neonatology, Hillel Yaffe Medical Center, 38100 Hadera, Israel

## Abstract

*Objective*. The objective of this study was to evaluate whether duration of breastfeeding is associated with a lower prevalence of metabolic syndrome in obese children. *Methods*. A retrospective analysis of obese children aged 3 to 18 years followed at a pediatric outpatient clinic at a single center between the years 2008 and 2012. The children were divided according to their breastfeeding duration: no breastfeeding, a short period of breastfeeding, and a long term breastfeeding. Also, they were divided into metabolic and nonmetabolic syndrome groups, based on physical examination and laboratory tests. *Results*. Out of 4642 children who visited the clinic, 123 were obese and were included in the study. About half of them matched the metabolic syndrome criteria. There was no correlation between the prevalence of metabolic syndrome and the duration of breastfeeding. Hypertension, abnormal low levels of HDL, high levels of HbA1c, and high fasting triglyceride levels were very common in our study population, yet no statistical significance was noted among the different breastfeeding groups. *Conclusion*. In this study, breastfeeding was not associated with a reduced risk for metabolic syndrome, compared with formula feeding, in children who are obese.

## 1. Introduction

Over the past decades, the prevalence of childhood obesity has grown worldwide [1–5]. Therapeutic interventions aimed at encouraging weight loss in obese children are costly and have had unsatisfactory long-term success rates [6]. Therefore, the identification of programs and strategies to prevent obesity more effectively has drawn much attention in recent years. Simple and cheap strategies without potential side effects are preferable. The concept that nutrition in infancy may have a long-term influence on the development of obesity first emerged in the 1960s with the pioneering work of McCance [7]. Recently, systematic reviews [8–11], based on population studies, have confirmed an association between breastfeeding and lower risk for developing obesity in adulthood. Owen et al. [12] reviewed studies which investigated the relation between breastfeeding and total cholesterol and low-density lipoprotein (LDL) cholesterol. One of their conclusions was that in adulthood, breastfed subjects had lower levels of total cholesterol than bottle-fed subjects. Another meta-analysis examined the relation between breastfeeding and blood pressure [13]. Mean systolic blood pressure was found to be lower at adulthood in breastfed participants than in bottle-fed ones. Another study [14] examined blood pressure in 216 adolescents who were born preterm and observed that those who were fed breast milk had a lower mean arterial blood pressure than those who were fed preterm formula. Regarding developing diabetes mellitus type 2 in adulthood, a systematic review [15] showed that breastfeeding, compared to formula-feeding, was associated with a lower risk for type 2 diabetes mellitus by almost 40%. Furthermore, it had a positive effect on fasting glucose and insulin levels. The association between breastfeeding and childhood obesity is uncertain, and results are inconsistent: some studies show a strong association between breastfeeding and childhood obesity [11, 13, 14] while others do not [16–18]. There are clinical studies that show that breastfeeding decreases the risk for adulthood obesity [8–11].

We have found no published studies testing the influence of the duration of breastfeeding on different aspects of the metabolic syndrome.

## 2. Materials and Methods

### 2.1. Design

In this study, we examined the association between breastfeeding duration and metabolic syndrome. Metabolic syndrome was defined by the presence of 3 out of 5 criteria: BMI ≥95th percentile for age and sex, hypertriglyceridemia (>120 mg/dL), high blood pressure (>95th percentile for age, sex, and height), high blood fasting glucose level (>100 mg/dL), and low HDL level (<45 mg/dL) [19]. In addition we examined the relationship between the breastfeeding duration and different laboratory tests (such as fasting blood cholesterol level, fasting blood triglycerides level, HbA1c, glucose, and insulin levels) and the systolic and diastolic blood pressures.

### 2.2. Patients' Selection

In this retrospective analysis, we included children aged 3–18 years, who visited the pediatric or pediatric nephrology outpatient clinics of a single center (Hillel Yaffe Medical Center, Harera, Israel) for hypertension and/or obesity (BMI > 95th percentile for age and sex), with or without metabolic syndrome, in the years 2008 to 2012.

### 2.3. Breastfeeding History

Duration of breastfeeding was noted and divided into three groups:

 Group 1: none (0–2 months),
 Group 2: short period (2–6 months),
 Group 3: long term (6 months and above) breastfeeding.

### 2.4. Physical Measurements

Blood pressure (BP) was measured using the oscillometric method. Hypertension (HTN) was defined as either systolic and/or diastolic blood pressure ≥95th percentile measured on three or more occasions. The degree of HTN is further delineated by the two stages [20]:

 Stage 1 hypertension: systolic and/or diastolic BP among the 95th percentile and 5 mmHg above the 99th percentile, or if in adolescents the BP exceeds 140/90 mmHg even when <95th percentile.
 Stage 2 hypertension: systolic and/or diastolic BP ≥99th percentile plus 5 mmHg.

Height and weight were measured using standardized procedures. Obesity was defined as body mass index (BMI) above 95% percentile for age and sex according to Centers for Disease Control and Prevention (CDC) chart [21].

### 2.5. Demographic and Ethnic Background

The children included (123) were of Jewish or Arab descents. All live around “Hadera” district in Israel.

### 2.6. Laboratory Assays and Methods

Laboratory tests were performed in the hospital laboratory. 12-hour fasting blood sampling was collected and tested for glucose, total cholesterol, LDL, HDL, triglycerides, insulin, and HbA1c levels. Definition for deviation values for blood test was high HbA1c: above 5.8%, impaired glucose tolerance: fasting glucose level above 100 mg/dL, insulin resistance: insulin level above 180 pmol/L, hypercholesterolemia: total cholesterol level above 200 mg/dL, hyperlipidemia: LDL level above 130 mg/dL, hypertriglyceridemia: TG level above 120 mg/dL, and low HDL: level below 45 md/dL [22].

### 2.7. Exclusion Criteria

Either insufficient laboratory data, or a presence of a diseases and/or a medicinal treatment that could affect metabolic functions and/or patient's weight (for example diabetes mellitus, Cushing syndrome, thyroid disorders, febrile illness at time the blood sampling, and systemic steroid usage).

### 2.8. Statistical Analysis

We used Student's *t*-test for unpaired samples to compare parametric data and the *χ* ^2^ test to compare nonparametric data between groups. Statistical significance was set at a *p* value < 0.05. All calculations were made using IBM SPSS software version 20 (International Business Machines Corp., New Orchard Road, Armonk, New York 10504). The study was approved by local board for human research. Informed consent was not needed as the study was retrospective, and collected data was unidentified.

## 3. Results

4642 children visited the pediatric or pediatric nephrology outpatient clinics during the study period. 168 (3.6%) children had BMI above 95% and were included. Out of them, 33 failed to provide information regarding breastfeeding and 12 could not define whether they had metabolic syndrome: for 7 of them lipid profile results were unavailable, and for the other 5 information regarding blood pressure follow-up was missing. Hence the final sample was 123 children (59 males and 64 females; 57 Arabs and 66 Jews). There were no statistically significant differences regarding sex or ethnic origin among the breastfeeding groups.

Metabolic syndrome: about half (47%) of the patients in this study matched the metabolic syndrome criteria [22]. These differences were not significant statistically in terms of the prevalence of metabolic syndrome among the subgroups (Table 1).

Table 2 summarizes the numbers and percents of children with hypertension, high HbA1c, high glucose level, high LDL level, low HDL level, high TC level, high insulin level, and high TG level in the whole group of the included children and in the groups of breastfeeding. There was no statistically significant difference among the subgroups regarding any of the above variables.

## 4. Discussion

This study aimed to find a relation between the duration of breastfeeding and the occurrence of metabolic syndrome in the pediatric population. The prevalence of the metabolic syndrome was about half of the obese children. This finding is similar to that of a previous study [22] that also demonstrates a high risk of metabolic syndrome in obese children.

We did not find any correlation between the prevalence of metabolic syndrome and the duration of breastfeeding.

Many similar studies were published in the last years and, like ours, have not found a relationship between breastfeeding and obesity in childhood [9, 17]. Nevertheless, some studies did find a correlation between breastfeeding duration and decreased incidence of metabolic syndrome later in life [8, 10, 11]. Thereupon, we can deduce that the matter is still controversial and that further investigation is needed regarding it.

In our study, we found no correlation between the duration of breastfeeding and abnormal concentrations of blood glucose and serum insulin.

There are only a few studies that tried to find a correlation between breastfeeding duration and type 2 diabetes mellitus. Furthermore, only in one study there was a positive correlation between breastfeeding duration and type 2 diabetes mellitus [15]. This subject is controversial, and further investigation is needed.

In our study, we found no significant difference among the breastfeeding groups regarding to the prevalence of hypertension. Searching medical literature, we found numerous studies regarding this issue, some of which have found reduced hypertension associated with breastfeeding [14, 23] and some have not [13].

Our study has several limitations; first, it is a small study in scope. In addition, it is a retrospective study. Moreover, patients' family history has not been taken into account and perhaps should have been.

## 5. Conclusion

In this study, breastfeeding was not associated with a reduced risk for metabolic syndrome, compared with formula feeding, in children who are obese.

## Figures and Tables

**Table 1:** Breastfeeding groups distributions of children with and without metabolic syndrome.

	All Patients	Metabolic syndrome	Non-metabolic syndrome
All	123	58 (47%)	65 (53%)
Group 1	57 (46.5%)	32 (56%)	25 (44%)
Group 2	37 (30%)	13 (35%)	24 (65%)
Group 3	29 (23.5%)	13 (45%)	16 (55%)
*p *		0.99	0.98

**Table 2:** Count of children with various abnormal results among the different subgroups.

	All	HTN	HbA1c	Glucose	LDL	HDL	TC	Insulin	TG
All	123	60 (49%)	35 (28.5%)	13 (10.5%)	14 (11%)	80 (65%)	12 (10%)	22 (18%)	35 (28.5%)
Group 1	57 (46.5%)	28 (49%)	16 (28%)	6 (10.5%)	4 (7%)	43 (75%)	5 (9%)	10 (17.5%)	23 (40%)
Group 2	37 (30%)	19 (51%)	12 (32%)	4 (10.5%)	6 (16%)	18 (48.5%)	3 (8%)	7 (19%)	6 (16%)
Group 3	29 (23.5%)	13 (45%)	7 (24%)	3 (10.5%)	4 (14%)	19 (65.5%)	4 (14%)	5 (17%)	6 (20.5%)
*p *		1.00	0.99	1.00	0.94	0.98	0.97	1.00	0.93

## Abbreviations

BMI: Body mass index
BP: Blood pressure
HbA1c: Hemoglobin A1c
HDL: High density lipoprotein
HTN: Hypertension
LDL: Low density lipoprotein
TC: Total cholesterol
TG: Triglycerides.
